# Puerarin as a Phytochemical Modulator of Gastrointestinal Homeostasis in Livestock: Molecular Mechanisms and Translational Applications

**DOI:** 10.3390/antiox14060756

**Published:** 2025-06-19

**Authors:** Jiehong Zhou, Jianyu Lv, Xin Chen, Tian Li, Jianzhong Shen, Zhanhui Wang, Chongshan Dai, Zhihui Hao

**Affiliations:** 1 Technology Innovation Center for Food Safety Surveillance and Detection (Hainan), Sanya Institute of China Agricultural University, Sanya 572025, China; 2 National Key Laboratory of Veterinary Public Health and Safety, College of Veterinary Medicine, China Agricultural University, Beijing 100193, China

**Keywords:** puerarin, intestinal health, intestinal barrier function, livestock and poultry production

## Abstract

The gut serves as the main site for nutrient digestion and absorption. Simultaneously, it functions as the body’s largest immune organ, playing a dual role in sustaining physiological equilibrium and offering immunological defense against intestinal ailments. Maintaining the structural and functional integrity of the intestine is paramount for ensuring animal health and productivity. Puerarin, a naturally derived isoflavonoid from the *Pueraria* species, exhibits multifaceted bioactivities, such as antioxidant, anti-inflammatory, antimicrobial, and immunomodulatory properties. Emerging evidence highlights puerarin’s capacity to enhance gut health in farm animals through four pivotal mechanisms: (1) optimization of intestinal morphology via crypt-villus architecture remodeling, (2) augmentation of systemic and mucosal antioxidant defenses through Nrf2/ARE pathway activation, and (3) reinforcement of intestinal barrier function by regulating tight junction proteins (e.g., ZO-1, occludin), mucin secretion, intestinal mucosal immune barrier, the composition of microbiota, and the derived beneficial metabolites; (4) regulating the function of the intestinal nervous system via reshaping the distribution of intestinal neurons and neurotransmitter secretion function. This review synthesizes current knowledge on puerarin’s protective effects on intestinal physiology in farm animals, systematically elucidates its underlying molecular targets (including TLR4/NF-κB, MAPK, and PI3K/Akt signaling pathways), and critically evaluates its translational potential in mitigating enteric disorders such as post-weaning diarrhea and inflammatory bowel disease in agricultural practices.

## 1. Introduction

The intestine fulfills a dual physiological mandate: it functions as the principal organ for nutrient assimilation while simultaneously serving as the primary immunological interface against luminal and systemic pathogen incursion [1]. Consequently, intestinal homeostasis is intricately linked to systemic health and organismal vitality. The intestinal mucosal barrier system is composed of four coordinated components, i.e., the mechanical barrier (such as epithelial tight junctions), the biochemical barrier (such as mucins and enzymes), the immunological barrier (such as gut-associated lymphoid tissues-associated defenses), and the microbial barrier (such as commensal microbiota). They act collectively to prevent pathogenic infiltration and inhibit the translocation of luminal-derived endotoxins, microbial fragments, and immunogenic antigens [2,3]. Preservation of this barrier’s multidimensional integrity, encompassing both architectural continuity (via epithelial regeneration) and functional competence, constitutes a critical determinant in sustaining intestinal physiological equilibrium and preventing enteropathy-associated systemic dysregulation [4,5].

In intensive livestock production systems, animals are chronically exposed to multifactorial stressors, including pathogen infections, thermal stress, and premature weaning, which collectively damage the intestinal epithelial barrier and compromise growth performance [6]. Historically, antibiotics were widely used prophylactically against enteric disorders, but this could result in drug residue and antimicrobial resistance issues, causing public health risks [7]. Consequently, developing sustainable alternatives to antibiotics, such as probiotic formulations, phytogenic bioactive compounds, and immunomodulatory nutraceuticals, has become a strategic priority in animal husbandry [8].

Emerging as promising alternatives to antibiotics, plant-derived bioactive compounds have garnered significant research attention owing to their diverse botanical origins, non-residual safety profiles, and multi-target mechanisms of action [9]. Among these, *Pueraria lobata (Willd.)* Ohwi is a perennial leguminous vine indigenous to Asian ecosystems (*Pueraria thomsonii* and *Pueraria lobata*). It has a rich repository of phytochemicals, including isoflavonoids (such as puerarin, daidzein, daidzin, and puerarin-7-xyloside), amino acids, polysaccharides, and trace elements with demonstrated bioactivity [10]. As the principal bioactive constituent, puerarin (an 8-C-glycosylated isoflavone) exhibits multimodal pharmacological properties, such as reactive oxygen species (ROS) scavenging, antioxidative stress, anti-inflammatory, anti-infection, immune regulation, anti-aging, improving intestinal function and maintaining the balance of gut microbiota effects [11,12,13,14,15,16,17,18,19,20,21]. These activities highlight puerarin’s therapeutic versatility across multiple disease spectrums, with demonstrated efficacy in ameliorating cardiovascular dysfunction, diabetes, hepatic pathologies, infective diseases, cancer, and neurodegenerative diseases [22,23,24,25,26,27]. Recent studies indicate that puerarin supplementation can effectively improve intestinal function and increase feed conversion efficiency, meat quality metrics (e.g., intramuscular fat deposition), and enteroprotective efficacy, finally improving livestock productivity [28,29,30]. For instance, Li et al. showed that dietary puerarin supplementation can markedly reduce the intestinal injury and oxidative stress in the small intestines of diquat-challenged piglets [31]. Wu et al. showed that puerarin supplementation at 0.5 mg/kg body weight (dissolved in a liquid milk replacer) for nine days can effectively reduce oxidative stress and inflammatory response, promote beneficial intestinal microbiota, and improve the function of the mucosal barrier in the intestine tissues of neonatal piglets, ultimately reducing morbidity caused by porcine epidemic diarrhea virus (PEDV) infection [21]. In this review, we summarized the current evidence on puerarin supplementation on its beneficial impact on animal intestinal health. Additionally, the underlying regulatory mechanisms in improving animal intestinal morphology, enhancing intestinal antioxidant capacity, and preserving the intestinal mucosal barrier were also discussed. Collectively, this review provides a comprehensive perspective for the intestinal health regulatory effects of puerarin in livestock, and it will promote its conversion and application in livestock production systems.

## 2. The Physicochemical Properties of Puerarin

**Figure 1:**
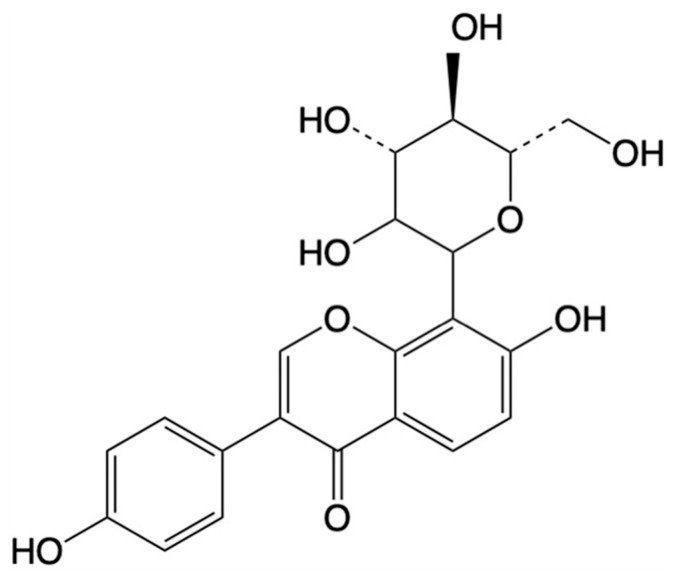
Chemical structure of puerarin.

Puerarin (also known as daidzein-8-C-glucoside) (Figure 1), is a predominant isoflavonoid in the root of *Pueraria lobata (Willd.)* Ohwi presents unique molecular characteristics that govern its pharmacokinetic behavior [32]. Pharmacopeial standards mandate ≥13% puerarin content in *Pueraria lobata*’s root extracts. With the molecular formula C_21_H_20_O_9_ (with a molecular weight of 416 Da), this crystalline compound exhibits pH-dependent solubility: freely soluble in methanol, sparingly soluble in ethanol, and poorly soluble in water (it is about 2.46 g/L at 25 °C) due to its amphiphilic nature arising from the hydrophobic isoflavone core and hydrophilic C-8 β-D-glucopyranosyl moiety [33,34,35]. The C-glycosidic linkage confers metabolic stability against β-glucosidase hydrolysis, a critical advantage over O-glycosylated analogs [27,36].

## 3. Absorption and Metabolism of Puerarin

Puerarin’s pharmacokinetics are characterized by limited absorption, broad tissue distribution, multi-phase metabolism, and enterohepatic-dependent elimination [37]. While negligible gastric absorption occurs, intestinal uptake in the duodenum and jejunum is rapid yet hindered by P-glycoprotein (P-gp) efflux and its low water solubility and permeability, resulting in the poor oral bioavailability (~7%) of puerarin [38]. However, the lower oral bioavailability (less than 1%) was also observed following oral higher dosing. This may be corrected by its low dissolution state in gastrointestinal fluid [38]. The poor intestinal absorption also resulted in a short half-life (*t*_1/2_) and low peak plasma concentration (*Cₘₐₓ*) of puerarin [39,40]. Therefore, selecting appropriate strategies to improve the physicochemical properties of puerarin is of great significance for further enhancing its oral bioavailability and promoting the development of its oral formulations. Several formulation innovations and drug delivery systems, such as nanoemulsions, phospholipid complexes, and solid self-microemulsifying, can effectively improve the oral bioavailability of puerarin in animal models [41,42,43]. For instance, Wu et al. reported that microemulsion-based puerarin exhibited a 15.8-fold increase in relative bioavailability compared to a suspension in mice [44]. Tang et al. prepared puerarin-loaded oil-in-water- and water-in-oil-microemulsions, which significantly increased the solubility to 11.3 and 23.1 mg/mL, respectively; and the oral relative bioavailability both increased 2-fold, compared to the puerarin suspension administration in rats [45]. Tu et al. prepared puerarin nanocrystals and improved the oral bioavailability of puerarin. In a rat model, the area under the curve (AUC) of the puerarin nanocrystals was 7.6-fold that of puerarin suspension, with an absolute bioavailability of 21.4% [46]. A systematic induction and review of the **drug delivery systems of** puerarin have been carried out by Zhang [37].

Following absorption, puerarin can distribute extensively to the heart, liver, kidney, lung, stomach, bone marrow, and mammary gland [38]. Although the concentration is limited, puerarin can still penetrate the blood–brain barrier (BBB) at pharmacologically relevant levels, and the glucose transporter type 1 (GLUT1) transporter promotes pharmacology-related BBB permeability [47,48,49]. Wu et al. found that co-administration with borneol or α-asarone (both at 25 mg/kg body weight) amplifies brain exposure of puerarin in rats, the AUC_0–12_ increased from 48.1  ng/mL·h to 64.8 ng/mL·h  or 86.0  ng/mL·h, respectively, thereby enhancing its targeting efficacy for cerebral ischemia-induced brain damage [47,50]. In the study of Anukunwithaya et al., *C_max_* of puerarin was reached within 5 minutes when rats were orally administered with 5 or 10 mg/kg body weight, and systemic clearance occurred at 8–12 h [38]. Several studies also showed that oral administration of a puerarin suspension resulted in C_max_ at 0.45–5.00 hours [35,51,52,53].

Extensive studies on puerarin metabolism in vitro and in vivo have revealed pathways including oxidation, glucuronidation, sulfation, creatinine conjugation, and gut microbiota-mediated metabolic transformation [38]. The major hydrolysis metabolite of puerarin was identified as daidzein, which was formed by cytochrome P450 proteins (CYP 450s) in the liver microsomes, and subsequently reduced to dihydrodaidzein and equol. Daidzein could also form daidzein-4′,7-di-O-sulfate and daidzein-4′-O-sulfate through the sulfation progress [54]. Glucuronides were the main metabolites of puerarin and were excreted in the urine and feces [38]. The glucuronide conjugates of puerarin, such as puerarin-7-O-glucuronide and puerarin-4′-O-glucuronide, were the main metabolites of the conjugation reaction [38]. It has been reported that seven UDP-glucuronosyl-transferase (UGT) isoforms (e.g., UGT1A1, 1A9, 1A10, 1A3, 1A6, 1A7, and 1A8) can catalyze the conversion of puerarin to form two water-soluble metabolites, i.e., puerarin-7-O-glucuronide and puerarin-4-O-glucuronide [55,56,57]. It has been demonstrated that puerarin-7-O-glucuronide can also contribute to potent activities in treating cardiovascular diseases, which is similar to its parent compound, puerarin [58]. Additionally, based on the data in rat liver and intestine, the metabolic profiles of puerarin were similar in liver and intestine, with no metabolic regioselectivity [37].

After absorption in the intestine, puerarin is rapidly excreted through urine and feces, mainly in the form of gluconates [37]. It is mainly excreted in urine, and approximately 50% and 15% of the intravenous dose is excreted via its gluconate metabolites through the urethra and the feces, respectively [38]. This process can be influenced by multiple factors, such as water solubility, bioavailability, gender, and pathological condition [37]. In addition, the pharmacokinetic characteristics of puerarin showed marked differences in its absorption and excretion patterns in vivo between *Pueraria lobata*’ root extract and pure puerarin after oral administration [59]. These findings suggest that puerarin’s metabolism is highly susceptible to external factors, necessitating further investigation into its complex metabolic pathways.

**Figure 2:**
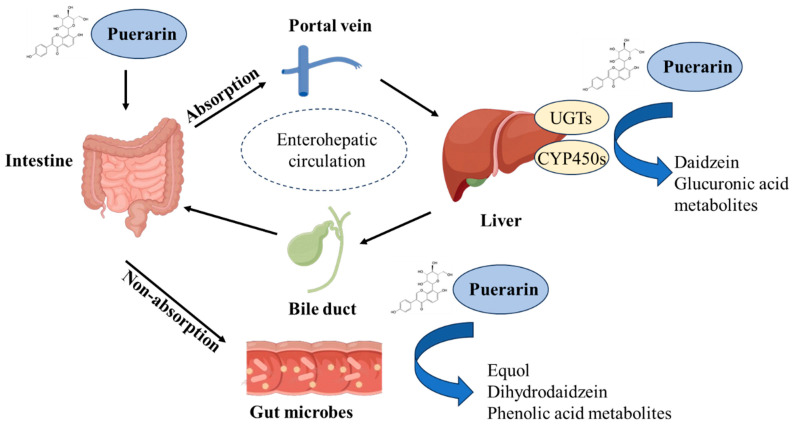
The absorption of puerarin and its metabolic pathways in the intestine and liver tissues. UGTs, UDP-glucuronosyltransferases; CYP450s, cytochrome P450s.

Correctly, puerarin undergoes enterohepatic recycling following oral administration. It is absorbed in the small intestine via glucose transporters and metabolized in the liver during first-pass effects by UGTs and sulfotransferases (SULTs) into sulfated and glucuronidated derivatives. A fraction of these metabolites is excreted into bile and is then hydrolyzed back to the original compound by gut microbiota, thereby initiating enterohepatic recycling. For example, gut microbiota-derived bioactive metabolite daidzein can be reabsorbed into liver via the portal vein, then provide a hepatoprotective effect (shown in Figure 2) [27,37]. The enterohepatic recycling mechanism underlies puerarin’s sustained efficacy and interpatient variability, highlighting its role as a dual-edged determinant for precision dosing strategies.

## 4. The Regulatory Effect of Puerarin on Intestinal Health of Farm Animals

### 4.1. Effects of Puerarin on Intestinal Morphology

The villus height (VH), crypt depth (CD), and VH/CD ratio serve as quantitative anatomical indices for evaluating intestinal mucosal integrity and digestive-absorptive capacity. In the small intestine, an increase in villus height (VH) expands the luminal surface area, enhancing nutrient absorption. Meanwhile, a decrease in crypt depth (CD) indicates improved epithelial regeneration efficiency [60]. Intestinal stem cells (ISCs) residing in the crypt base undergo continuous proliferation and upward migration along the crypt-villus axis, differentiating into functional enterocytes to maintain homeostatic turnover—a critical balance between apoptosis and regeneration that underpins mucosal barrier function [61]. Mechanistic studies reveal puerarin’s therapeutic potential in preserving intestinal morphology under pathological stress. For instance, Dong found that the oral administration of puerarin at 5 mg/kg body weight for six days can enhance the values of VH and the ratio of VH/CD, and reduced oxidative stress and inflammation in piglets infected with Enterotoxigenic *Escherichia coli* (ETEC), thereby mitigating intestinal morphological damage and improving the production performance (such as growth rate and feed conversion rate) [62]. In another study, dietary supplementation with puerarin at 750 mg/kg feed in yellow-feathered broilers (*Gallus gallus* domesticus) can effectively reverse oxidized soybean oil-induced ileal atrophy, restoring duodenal VH to 428 μm (vs. 312 μm in controls) and VH/CD to 2.9 (vs. 1.7 in controls) [63]. The equilibrium of intestinal morphology is intricately linked to the balance between apoptosis and proliferation of intestinal cells. Proliferating cell nuclear antigen (PCNA) serves as a marker for cell proliferation [64,65]. Li et al. indicated that incorporating 0.1% puerarin into the diet of weaned piglets can increase the proportion of PCNA-positive cells in the intestinal mucosa, thereby promoting intestinal epithelial cell proliferation and alleviating the intestinal and ileal morphological damage induced by diquat [31].

These findings collectively demonstrate that puerarin stabilizes crypt-villus dynamics by reducing oxidative stress-induced apoptosis (via ROS scavenging) and promoting epithelial renewal (via Wnt/β-catenin activation), thereby preserving intestinal architectural homeostasis under diverse pathological insults.

### 4.2. Effect of Puerarin on Intestinal Antioxidant Capacity

Modern high-density livestock operations expose animals to cumulative oxidative stressors—heat stress, environmental toxins, infectious diseases, and weanling stress—that overwhelm endogenous antioxidant defenses. This redox imbalance drives pathological ROS accumulation, culminating in macromolecular oxidative lesions across tissues [66,67].

Currently, exogenous supplementation of antioxidants represents a critical strategy for enhancing systemic antioxidative capacity and mitigating intestinal oxidative stress in animal health management. A recent study showed that oral administration of puerarin at 5 mg/kg body weight for six days can significantly augment enzymatic defenses in ETEC-challenged piglets, as evidenced by significantly increased total superoxide dismutase (T-SOD) and catalase (CAT) activities in the ileum tissues of ETEC-challenged piglets [21]. In another study, it was found that puerarin supplementation improved antioxidant activity by significantly increasing the activity of total antioxidant capacity, glutathione peroxidase (GSH-Px), and CAT, while decreasing malondialdehyde (MDA) levels in the serum, jejunum, and ileum in aged laying hens, finally improving intestinal morphology with increased villus height and the ratio of villus height to crypt depth [30].

Nuclear factor erythroid 2-related factor 2 (Nrf2), encoded by the NFE2L2 gene, belongs to the Cap’n’Collar (CNC) basic leucine zipper transcription factor family. These evolutionarily conserved regulators are ubiquitously expressed in eukaryotic organisms and function as master regulators of cytoprotective gene networks that mediate adaptation to environmental stressors [68]. Upon activation, Nrf2 translocates to the nucleus, where it binds to antioxidant response elements (AREs) and triggers the expression of detoxifying enzymes such as heme oxygenase-1 (HO-1), NAD(P)H quinone oxidoreductase 1 (NQO1), glutamate cysteine ligase (GCLC), SOD, CAT, and glutathione S-transferase (GST) [69]. Puerarin has been identified as a potent Nrf2 activator, functioning through the Keap1-Nrf2-ARE signaling axis to upregulate downstream cytoprotective genes including HO-1, SOD, CAT, and NQO1, and GCLC [11,70,71,72]. Consistently, Nrf2 knockdown or knockout significantly reduces puerarin-induced oxidative stress damage to various environmental toxins or drugs in tissues or cells [11,70,73]. Li et al. demonstrated that dietary inclusion of 0.1% puerarin markedly upregulated the expression of Nrf2 protein in both jejunal and ileal mucosa tissue of piglets, then further induced the transcriptional expression of downstream gene-mediated protein expression (e.g., HO-1, GSH-Px, and CAT) [31]. In a separate intervention, oral administration of puerarin (0.5 mg/kg body weight) induced a marked increase in the levels of Nrf2 mRNA in the intestinal tissues, then significantly increased the levels of T-SOD, GSH-Px, and CAT activities, and finally, effectively reduced the damage in PEDV-infected piglets [21].

**Figure 3:**
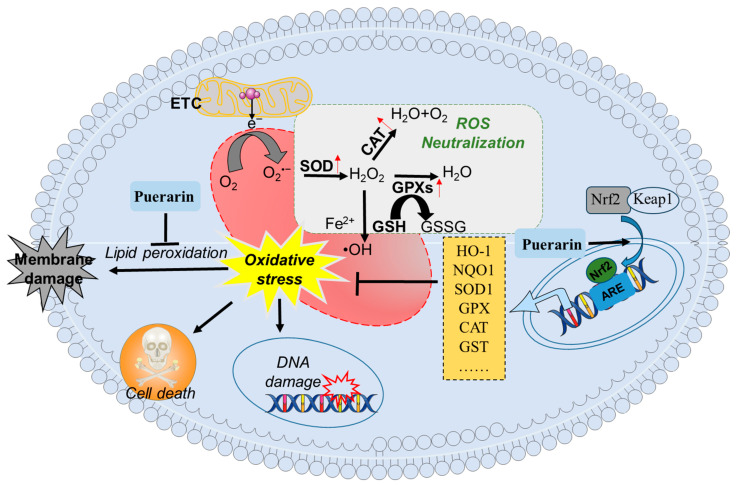
A working model of puerarin protecting against oxidative stress in the intestinal tissues. GSSG, oxidized glutathione; SOD, superoxide dismutase; SOD, superoxide dismutase; ROS, reactive oxygen species; H_2_O_2_, hydrogen peroxide; •OH, hydroxyl radical; O_2_^•−^, superoxide anion; O_2_^•−^, superoxide anion; Nrf2, nuclear factor erythroid 2-related factor 2; NQO1, NAD (P)H quinone oxidoreductase 1; NQO1, NAD (P)H quinone oxidoreductase 1; HO-1, heme oxygenase 1; GSH, glutathione; CAT, catalase; ETC, electron transport chain; ARE, antioxidant response element.

Collectively, these findings demonstrate that puerarin supplementation alleviates stress-induced intestinal damage in animals by inhibiting lipid peroxidation, enhancing antioxidant enzyme activity, or directly activating the Nrf2-mediated antioxidant defense pathway (Figure 3).

### 4.3. Effect of Puerarin on Intestinal Barrier Function

**Figure 4:**
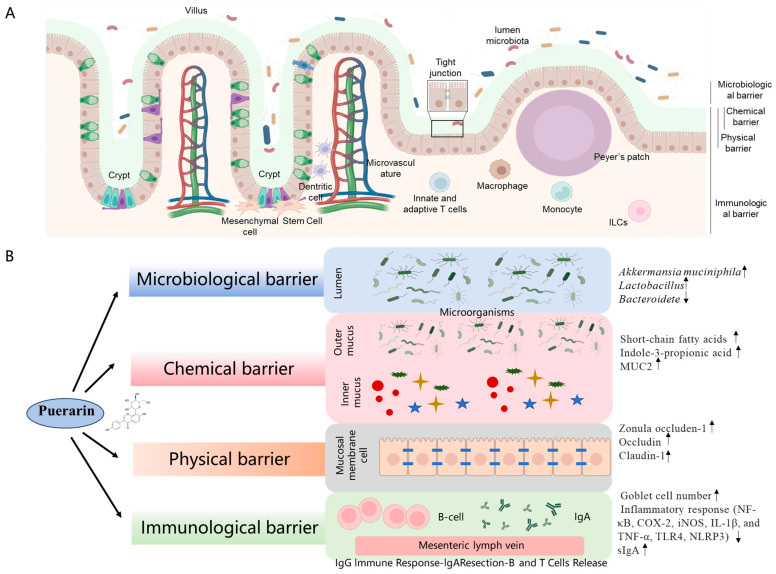
(**A**), intestinal barrier structure. (**B**), a working model of puerarin in maintaining the intestinal barrier function in the intestinal tissues. Puerarin supplementation can integrate and regulate the microbiological barrier (e.g., increase the levels of *Akkermansia muciniphila* and *Lactobacillus*, and decrease the levels of *Bacteroidete*), chemical barrier (e.g., increase the levels of short-chain fatty acids, indole-3-propionic acid, and secreted MUC2), physical barrier (improve the tight junctions in intestinal epithelial cells via upregulating the expression of zonula occluden-1, occludin, and claudin-1 proteins), and immunological barrier (e.g., increase the number of goblet cells and the levels of sIgA, and reduce the inflammatory response), and synergistically maintain the intestinal barrier function.

The intestinal barrier represents a sophisticated multidimensional defense system comprising four interactive components, including the mechanical barrier (also called the physical barrier), the chemical barrier, the immunological barrier, and the microbiological barrier [74,75,76]. Puerarin supplementation can integrate and regulate these barriers synergistically to prevent systemic dissemination of luminal pathogens and toxins through mechanisms including mucin secretion, tight junction regulation, IgA-mediated immune surveillance, and commensal microbiota modulation (shown in Figure 4). The detailed actional mechanisms will be discussed in the following sections.

#### 4.3.1. Intestinal Microbiological Barrier

The intestinal microbial barrier, consisting of the intestinal microbiota, forms a relatively stable ecosystem through mutual balance. The gut microbiota is instrumental in breaking down indigestible dietary fiber into short-chain fatty acids (SCFAs) within the gastrointestinal tract; these SCFAs provide energy, inhibit pathogenic microorganisms, and enhance the intestinal mucosal barrier [77]. Disruptions in the gut microbiota can impair mechanical barriers and intestinal morphology, precipitating intestinal inflammation [78]. The gut microbiota analysis revealed a significant reduction in Bacteroidetes coupled with *Akkermansia muciniphila* (AKK) enrichment in the puerarin-treated intestinal tissue, suggesting enhanced mucin utilization capacity. Notably, puerarin demonstrated selective elevation of fecal indole-3-propionic acid (IPA) concentrations while maintaining indoleacrylic acid and butyrate at physiological baselines [79]. Moreover, IPA can activate the aryl hydrocarbon receptor (AhR) signaling pathway, which can regulate barrier integrity and immune function by influencing the expression of tight junction proteins and modulating inflammatory responses [80]. It indicated that the marked upregulation of IPA in the intestinal tissues induced by puerarin may provide protection via the activation of AhR [79,81]. Intravenous administration of puerarin increased the number of lactic acid bacteria in the cecum and colon of ETEC-infected piglets, alleviating bacterial dysbiosis. Furthermore, puerarin enhances the relative abundance of Lactobacillus in the gut of mice with irritable bowel syndrome and decreases the relative abundance of *Desulfovibrionaceae* [82]. In addition, research also showed that oral administration of puerarin increases the relative abundance of AKK bacteria in mice under normal physiological conditions, colitis rats, and obese mice [83,84]. AKK bacteria can stimulate intestinal stem cell proliferation and IL-10 secretion, repairing *Salmonella*-induced intestinal damage [85]. Studies revealed that puerarin exerts a direct inhibitory effect on ETEC, significantly diminishing the expression of ETEC subunit proteins and virulence factors, and curbing the secretion of exotoxins [86]. Consistently, it also found that the intervention of silymarin and polyherbal extract (it includes silymarin, salvianolic acids B, and puerarin) significantly increased the levels of *Akkermansia* and *Blautia*, and reduced the levels of *Clostridium* in the intestinal tissues, then improved high-fat diet-induced liver injury [87].

In short, puerarin can promote microecological reprogramming via enriching AKK while suppressing *Bacteroidete*, synergistically elevating IPA to AhR signaling, and finally contributing to its protective effect on the dysfunction of the intestinal barrier. Moreover, this dual mechanism, i.e., enhancing the integrity of the mucus layer through goblet cell hyperplasia and simultaneously optimizing the ecology of mucinophilic microbiota, demonstrates puerarin’s therapeutic potential as a nutraceutical agent for precisely restoring the intestinal barrier by modulating host–microbiota crosstalk.

#### 4.3.2. Intestinal Chemical Barrier

The intestinal chemical barrier, a multifunctional defense system comprising enzymatic secretions, antimicrobial peptides (e.g., defensins), and microbiota-derived metabolites (e.g., SCFAs and IPA), relies critically on the mucin 2 (MUC2)-mediated mucus layer (>300 μm) to prevent pathogen adhesion (such as *Salmonella*) while maintaining luminal pH homeostasis (6.0–7.4) [88,89,90,91]. Notably, SCFAs, mainly including acetate, propionate, and butyrate, are metabolites produced during the bacterial fermentation of dietary fiber in the intestinal tract. Butyrate is the principal substrate and energy source for colonocytes and provides at least 60–70% of colonic mucosal energy requirements [92]. Concurrently, SCFAs can reinforce barrier integrity by energizing colonocytes (i.e., ~70% ATP from butyrate β-oxidation) and upregulating Claudin-5/MUC2 expression via GPR43/HDAC modulation [93]. It has been demonstrated that physiological concentrations of SCFAs immediately suppress colonic epithelial permeability and reduce the invasion of exogenous toxins and pathogens [93]. Several studies have illustrated that puerarin can enhance the chemical barrier through three synergistic mechanisms: (1) stimulating goblet cell MUC2 secretion via Ca^2+^/PKC-dependent exocytosis, (2) elevating protective metabolites, including fecal IPA and butyrate through bifidobacterium enrichment, and (3) suppressing inflammation via IPA-mediated NF-κB inhibition, downregulating the expression of tumor necrosis factor-α (TNF-α), interleukin-8 (IL-8), and IL-6 [61,94,95]. Consistently, Liu et al. demonstrated that oral administration of 10 mg/kg body weight puerarin can increase the activity of sucrase, protease, and amylase in the intestinal tract of mice, then promote the digestion and metabolism of carbohydrates [96]. Collectively, these actions establish puerarin as a multimodal enhancer of chemical barriers, integrating mucus reinforcement, regulation of microbial metabolites, and optimization of digestive processes to strengthen intestinal mucosal defenses.

#### 4.3.3. Intestinal Mechanical Barrier

Tight junctions (TJs), the principal intercellular junctional complexes of the intestinal mechanical barrier, are dynamically regulated by transmembrane proteins (occludin, claudin family) and scaffolding proteins (Zonula occludens, ZO). These junctions function to seal intercellular spaces and selectively facilitate the passage of nutrients, electrolytes, and water from the intestinal lumen through the intestinal mucosa, while simultaneously preventing the infiltration of harmful substances [97]. A compromise in the integrity of this mechanical barrier results in increased intestinal permeability, allowing pathogens from the intestinal lumen to translocate into the submucosal tissue. This translocation activates the immune system, prompting the release of inflammatory cytokines and leading to intestinal inflammation [98,99]. In rat models of colitis induced by dextran sulfate sodium (DSS) and 2,4,6-trinitrobenzenesulfonic acid (TNBS), puerarin has been demonstrated to enhance TJ mRNA and protein expression levels, thereby restoring the integrity of the mechanical barrier [15]. Dietary supplementation with 1% puerarin has been shown to significantly elevate the mRNA expression levels of occludin and E-cadherin in the jejunum and ileum of piglets under oxidative stress [100]. Similarly, the supplementation of 750 mg/kg puerarin in the diet markedly enhances the mRNA expression levels of occludin, claudin-1, and ZO-1 within the intestinal tract of broiler chickens, thereby mitigating the mechanical barrier damage caused by oxidized soybean oil [38,99]. Additionally, puerarin has been shown to ameliorate intestinal mechanical barrier damage and reduce intestinal permeability in obese mice induced by a high-fat diet and in ovariectomized rats [83,101]. These findings suggest that puerarin plays a crucial role in restoring the integrity of the intestinal mechanical barrier in animals subjected to stress by upregulating the expression of TJ-relative proteins, thus safeguarding intestinal health (Figure 4).

#### 4.3.4. Intestinal Mucosal Immune Barrier

The intestinal mucosal immune barrier constitutes a sophisticated interface integrating structural, immunological, and neural components to maintain gastrointestinal homeostasis. Central to this system, the gut-associated lymphoid tissue (GALT) orchestrates immune surveillance through specialized structures including Peyer’s patches and mesenteric lymph nodes, coordinated by regulatory T cells that enforce immune tolerance and reduce the inflammatory response [102,103,104]. The epithelial barrier, comprising enterocytes and goblet cells, forms a selective physical blockade while actively participating in immune signaling through pattern recognition receptors [105]. Disruptions in the mucosal immune barrier can lead to increased intestinal permeability, allowing translocation of bacteria and their products, which can trigger systemic inflammation and contribute to the pathogenesis of various diseases, including inflammatory bowel disease, bacterial diarrhea, and virus infection-related gut dysfunction [106,107]. Li et al. found that puerarin supplementation via oral administration at 200 mg/kg body weight for 14 days can markedly restore mucus barrier integrity by upregulating mucin synthesis (e.g., upregulated the expression of MUC2 and MUC4 expression) and increasing goblet cell density in intestinal barrier dysfunction in rats exposed with 2,4,6 trinitrobenzene sulfonic acid (TNBS)-indued intestinal barrier dysfunction in rats [79].

The dysfunction of the intestinal mucosal immune barrier is a critical aspect of various inflammatory diseases, where the integrity of epithelial and endothelial barriers is compromised, leading to increased permeability and subsequent inflammation [108]. This dysfunction is often mediated by complex inflammatory pathways involving a variety of cellular and molecular mechanisms. One of the key players in barrier dysfunction is the nuclear factor kappa B (NF-κB) signaling pathway. Activation of NF-κB leads to the transcription of genes (such as cyclooxygenase-2 [COX-2], nitric oxide synthase [iNOS], IL-1β, and TNF-α) involved in inflammation and immune responses, contributing to the disruption of tight junction proteins and increased barrier permeability [109]. Studies have demonstrated that puerarin supplementation can effectively inhibit the transcriptional expression of NF-κB, then markedly downregulate the pro-inflammatory mediators such as COX-2, iNOS, IL-1β, and TNF-α, thereby alleviating DSS-induced colitis in mice [15]. Similarly, Wu et al. reported that puerarin supplementation can effectively inhibit the NF-κB signaling pathway in the ileum, decrease the levels of IL-1β, IL-6, and IL-8, and alleviate the damage of PEDV to the intestine [21,110]. In another study, it was reported that puerarin supplementation also inhibited the expression of NF-κB inflammatory signaling pathway-related genes in IPEC-J2 cells, reduced the number of ETEC adhesions, and mitigated ETEC-induced cell damage [111]. Consistently, puerarin supplementation can also markedly inhibit PEDV replication in both in vitro and in vivo settings and significantly reduce NF-κB activation-mediated inflammation response, effectively mitigating the decline in growth performance among piglets [21,110]. Zhou et al. found that dietary puerarin supplementation at 750 mg/kg feed via the diet can markedly inhibit the Toll-like Receptors 4 (TLR4)/NF-κB signaling pathway in the intestinal tract of broiler chickens, upregulate the expression of negative regulatory factors and anti-inflammatory cytokine genes, suppress the expression of pro-inflammatory cytokine genes, elevate the levels of secretory immunoglobulin A (sIgA), and ameliorate intestinal inflammation triggered by oxidized soybean oil [28]. These findings indicate that puerarin can effectively inhibit the inflammatory response to maintain the intestinal mucosal immune barrier via the inhibition of the NF-κB pathway.

Inflammasomes, particularly the NLRP3 inflammasome, play a crucial role in mediating inflammatory responses that lead to barrier dysfunction. Activation of the NLRP3 inflammasome results in the production of pro-inflammatory cytokines such as IL-1β, which can further exacerbate barrier disruption and inflammation [109,112]. Peng et al. discovered that puerarin activates the AMP-activated protein kinase (AMPK)/Sirtuin 1 (SIRT1) signaling pathway, inhibits the formation of NOD-like receptor protein 3 (NLRP3) inflammasomes, reduces IL-1β and IL-18 levels, and enhances the resistance of gastric epithelial cells to lipopolysaccharide (LPS)-induced apoptosis [113]. In addition, puerarin supplementation also exerts a mitigating effect on intestinal inflammation associated with obesity, food allergies, and irritable bowel syndrome [82,83,114]. Puerarin can also significantly increase the expression of TRPM5, a necessary protein for mucin secretion [81], but the precise molecular mechanisms are still unclear, and further investigations are required.

Of note, several studies showed that puerarin combined with tryptophan or its metabolites (e.g., IPA) can markedly improve the mucus layer barrier of intestinal tissues in dextran sodium sulfate-treated rats, which was mainly achieved by increasing the number of goblet cells and promoting the secretion of MUC2 [81]. This synergistic effect may also be related to the different regulatory effects of puerarin and tryptophan on the expression of TRPM5 protein in the intestinal tissues [81].

Collectively, puerarin orchestrates intestinal mucosal repair through the synergistic effects on mucosal reinforcement and anti-inflammatory circuitry. It can upregulate mucin synthesis and goblet cell density and dually inhibit the NF-κB and NLRP3 inflammasome pathways through AMPK/SIRT1 activation. These mechanistically stratified effects validate puerarin’s potential as a phytoceutical intervention for barrier-related pathologies ranging from infectious enteritis to metabolic-associated gut disorders.

### 4.4. Enteric Nervous System

The enteric nervous system (ENS) has a close interaction with the intestinal epithelial barrier and the surrounding immune cells and plays a crucial part in maintaining the homeostasis of the gastrointestinal tract [115]. It has been reported that the ENS is a key factor in the pathogenesis of various diseases, such as cancer, diabetes, and neurodegenerative diseases, and regulates gut barrier function and intestinal homeostasis [116].

The ENS is divided into two nerve plexuses. In the myenteric plexus, the cell bodies are located between the circular and longitudinal muscle layers of the intestine, and they coordinate the contraction and relaxation required for gastrointestinal peristalsis. Meanwhile, the cell bodies of the submucosal plexus are closer to the lumen and penetrate deep into the mucosa [116]. They coordinate secretion, absorption, and local blood flow in the intestinal tissues [116]. ENS can sense the stimuli from the lumen and respond with appropriate effector signals to promote digestion, movement, and host defense. It can also develop and influence the mucosal immune system and the gut microbiota, jointly regulating gut homeostasis [117]. For instance, Lactobacillus strains can directly regulate colonic motility by activating calcium-dependent potassium channels in the intrinsic sensory neurons of the ENS [118].

A recent study reported that puerarin exerts inhibitory effects on the motor dorsal nucleus–vagus nerve axis by binding to gamma-aminobutyric acid receptors containing alpha 1 subunit, thereby controlling the length of microvilli in the jejunum to regulate the absorption of fat by the intestine [119]. In another study, it was found that puerarin treatment can significantly decrease the nitric oxide, acetylcholine, and serotonin levels, reduce the contraction response of the intestine, and thereby improving diarrhea-predominant irritable bowel syndrome in rats [120]. Huang reported that puerarin supplementation can upregulate the proportions and quantities of inhibitory neurons (e.g., neuronal nitric oxide synthase [nNOS]- and vasoactive intestinal peptide [VIP]- immunoreactive myenteric neurons) and downregulate proportions and quantities of the excitatory neurons (e.g., neocortical choline acetyltransferase [ChAT]- and substance P [SP] immunoreactive myenteric neurons) in myenteric plexus, maintaining the homeostasis of colonic interneurons and smooth muscle contraction function, and finally, improving intestinal function in rat models of diarrhea-predominant irritable bowel syndrome [121].

Collectively, puerarin can regulate the intestinal nervous system by affecting the number and proportion of intestinal neurons and the secretion of neurotransmitters, thereby regulating intestinal contraction, nutrient absorption, and the response to harmful exogenous substances. However, the precise mechanism still needs further research.

### 4.5. Others

Intestinal stem cells are the driving force behind intestinal epithelial renewal, and when damaged, they quickly replenish lost stocks to support epithelial repair. This plasticity mechanism is crucial for intestinal health [122]. Tao et al. found that puerarin supplementation can increase Paneth cell numbers and stimulate the expression of Wnt ligand Wnt3a and Lgr5 and PCNA in the intestinal tissues of sleep disorder-treated aged mice [123]. Of note, Lgr5 serves as a marker for intestinal stem cells and increased levels of PCNA signify cell proliferation. This information indicated that puerarin supplementation can enhance the ability of intestinal damage repair via modulating the number and functions of intestinal stem cells [123]. Additionally, it also reported that puerarin supplementation can increase the expression of phosphorylation-extracellular signal-regulated kinase (p-Erk) and p-Akt protein, and improved the wound healing ability [124]. It is known that the activities of Erk and Akt play a critical role in intestinal modification, cell proliferation, immune regulation, and stress resistance [125]. These modulations may also contribute to its effects on intestinal repair. The precise molecular mechanisms still need more investigation.

## 5. Pharmacological Modulation of Production Performance in Livestock by Puerarin

### 5.1. Poultry Health

The application of puerarin has gained attention in recent years for its potential benefits in poultry production. One of the primary benefits of puerarin in poultry production is its ability to improve growth performance. Puerarin can enhance feed intake and conversion efficiency, leading to improved weight gain and overall growth in poultry. For instance, Lu Yu et al. discovered that adding 10 mg/kg of total flavonoids from *Pueraria lobata* (with a puerarin content of 79%) in the feed can significantly enhance the average daily weight gain of broiler chickens and decrease the feed conversion ratio via the enhancement of mucin secretion and the upregulation of short-chain fatty acids derived from gut microbiota [63]. The use of natural additives to bolster the immune system and reduce the prevalence of pathogens is a growing area of interest in poultry science. For instance, the inclusion of probiotics, prebiotics, and other natural compounds in poultry diets has been shown to improve gut health and immune responses, thereby reducing the incidence of diseases [126]. Puerarin, with its antioxidant and anti-inflammatory properties, could play a similar role in enhancing the immune resilience of poultry, potentially reducing the need for antibiotics and other chemical interventions.

In addition to growth optimization, puerarin may also contribute to disease resilience in poultry. For example, it was shown that puerarin treatment of 100 mg/kg in the diet can markedly improve disease resistance by increasing antioxidant enzyme activity, reducing lipid peroxidation and inflammatory response, and improving caspase-8-mediated cell apoptosis in broiler chickens infected with *Salmonella* [63,127]. Zhou et al found that puerarin supplementation at 750 mg/kg body weight via the diet significantly increased the serum immunoglobulin content, reduced the HMGB1, MAPK, and TLR4 signaling pathway-mediated inflammatory response, and mitigated intestinal barrier damage caused by oxidized soybean oil in the intestine tissue of broiler chickens [28].

Furthermore, puerarin’s potential role in modulating the gut microbiota is another avenue through which it could enhance poultry health. The gut microbiome is crucial for nutrient absorption and immune function, and its modulation through dietary interventions can lead to improved health outcomes in poultry. Research has highlighted the importance of maintaining a balanced gut microbiota for optimal poultry health and performance, with strategies such as the use of bacteriophage cocktails showing promise in this regard [128]. Gut microbiome analysis reveals a dual effect: suppression of *Enterobacteriaceae* and enrichment of butyrate-producing *Clostridium* clusters IV/XIVa, synergistically enhancing barrier function. This finding indicated that puerarin could contribute to this balance, supporting a healthy gut environment that fosters better nutrient utilization and disease resistance.

In short, puerarin supplementation can effectively improve intestinal health by reducing inflammatory response and remodeling gut microbiota. It presents a promising opportunity to enhance growth optimization and disease resilience in poultry production by improving intestinal health. By improving growth performance, bolstering immune function, and supporting gut health, puerarin could contribute to more sustainable and efficient poultry farming practices. Further research is needed to fully elucidate its mechanisms of action and to optimize its use dosage in commercial poultry operations, but the potential benefits make it a valuable area of exploration for the future of poultry production.

### 5.2. Porcine Health

Currently, research on puerarin in pig production is somewhat limited, with a primary focus on its impact on stressed piglets. Zeng et al. reported that puerarin supplementation at 5 mg/kg body weight can markedly enhance the antioxidant capacity and alleviate intestinal inflammation in *Escherichia coli* K88-infected piglets [129]. Furthermore, oral administration of 0.5 mg/kg puerarin has been shown to mitigate intestinal inflammation and oxidative stress caused by PEDV challenge [21,110]. Li et al. discovered that incorporating 0.1% puerarin into the feed can ameliorate the decline in growth performance of piglets infected with the enemy grass fast attack toxin [31]. Liu et al. found that *Pueraria lobata* root crude extract supplementation at 0.5 kg/ton feed in the diet can improve the gut microbial diversity of finishing pigs, enhancing the intestinal digestion and absorption of nutrients [130].

Collectively, these studies suggested that puerarin not only enhanced the intestinal and general health of stressed piglets and their stress resistance but also exerted a positive effect on the growth of piglets and fattening pigs under normal physiological conditions.

### 5.3. Cattle Health

Puerarin’s rumen-modulating properties stem from its bifunctional capacity as both a fibrolytic enhancer and thermoresistant. Nie et al. discovered that the supplementation of 0.04% puerarin in beef cattle feed can significantly enhance rumen fermentation patterns and increase the degradation rate of feed nutrients and rumen bacterial protein synthesis [131]. This information indicates that puerarin supplementation via the diet can promote the growth of beneficial bacteria that enhance fiber digestion via modulation of the microbial population in the rumen in cattle. Under heat stress conditions, the inclusion of 0.04% puerarin in beef cattle feed significantly enhanced the apparent digestibility of dry matter, crude protein, and neutral detergent fiber by 3.23%, 3.19%, and 2.19%, respectively [132]. This improvement in digestibility was accompanied by a significant increase in fecal microbiota diversity and the relative abundance of beneficial bacteria, which in turn improved the growth performance, immune function, and antioxidant capacity of beef cattle [132]. Several studies have shown that the addition of 400-900 mg/kg puerarin to feed can improve the digestion rate of nutrients in beef cattle by regulating rumen fermentation mode and microbial community structure, enhancing the antioxidant capacity of muscle tissues [133,134].

Collectively, these findings indicate that puerarin supplementation can effectively improve the intestinal microbiota and antioxidant capacity of cattle, thereby enhance their digestive ability and improving their productivity. Since cows are ruminants, their digestive systems may differ from those of other animals such as chickens or pigs. Therefore, their precise mechanisms still need further investigation.

Although these data suggest that supplementing with puerarin can partly improve the intestinal health of poultry, porcine, and cattle, thereby enhancing their production performance, it still has some limitations. For example, the sample size, experimental period, and scale in the above-mentioned studies are very limited and insufficient. In addition, current experimental studies on the dosage of puerarin use are diverse, with some studies using relatively high doses, which may increase costs. Therefore, a more in-depth, systematic, and comprehensive evaluation of the clinical efficacy of puerarin supplementation in intestinal infections is still highly needed.

## 6. Conclusions and Future Perspectives

As a pleiotropic phytochemical, puerarin was demonstrated to have multidimensional gastrointestinal protective efficacy based on its multimodal bioactivities, such as anti-inflammatory, antioxidant, regulating gut microbiota, and immune regulation. These mechanisms synergistically enhance intestinal villus architecture, reinforce mucosal barrier integrity, and stabilize gut–liver axis homeostasis, positioning it as a viable antibiotic-alternative feed additive for sustainable livestock production.

Moreover, the environmental and economic benefits of using natural additives like puerarin in livestock production cannot be overlooked. As the industry moves towards more sustainable practices, the reduction in antibiotic use and the implementation of natural growth promoters are becoming increasingly important. The use of puerarin aligns with these goals, offering a natural and potentially cost-effective alternative to traditional growth promoters and disease control measures. Importantly, a healthy gut can reduce harmful gas emissions from feces. It has been demonstrated that the essential microbes may effectively reduce the enteric methane emission and help to mitigate the global methane crisis [135].

To advance translational applications, three strategic research priorities emerge: (1) in terms of life-stage-specific pharmacology, we need to delineate dose–response relationships across poultry growth phases (broiler starter/grower/finisher) and lactating versus gestating swine models to optimize feeding regimens; (2) in terms of mechanistic investigation, the precise molecular targets puerarin in barrier regulation, particularly its interplay with TLR4 signaling and occludin phosphorylation dynamics, need to be further addressed. Some new technologies such as artificial intelligence, CRISPR-interference screening or multi-omics approaches may effectively accelerate our mechanism exploration; (3) in terms of bioavailability engineering, new formulations, such as β-cyclodextrin-encapsulated nano-formulations or others need to be investigated to overcome inherent solubility limitations; (4) in terms of safety assessments, the long-term endocrine effects and microbiota adaptation patterns caused by puerarin under industrial farming conditions should be further considered; (5) more systematic and comprehensive clinical studies (such as sufficient sample size, longer-term, and large-scale clinical trials) are still needed; (6) some investigations have shown that puerarin with its metabolites or other natural products may have a certain synergistic effect, thus maximizing its benefits for the intestinal health of livestock, which should be paid attention to in the future. By bridging the gap between growth promotion and disease prevention, with demonstrated benefits across poultry, swine, and ruminant systems, it promotes the translational applications to realize its full economic and ecological potential.

## Data Availability

All data in this article were obtained based on the author’s request to the corresponding authors.
